# *Ex vivo* susceptibility-weighted imaging anatomy of canine brain–comparison of imaging and histological sections

**DOI:** 10.3389/fnana.2022.948159

**Published:** 2022-09-02

**Authors:** Germain Arribarat, Benjamin Cartiaux, Samuel Boucher, Charles Montel, Hélène Gros-Dagnac, Yoann Fave, Patrice Péran, Giovanni Mogicato, Alexandra Deviers

**Affiliations:** ^1^ToNIC, Toulouse NeuroImaging Center, Université de Toulouse, INSERM, UPS, Toulouse, France; ^2^ToNIC, Toulouse NeuroImaging Center, Université de Toulouse, INSERM, UPS, ENVT, Toulouse, France

**Keywords:** susceptibility-weighted imaging, histology, canine, brain, atlas

## Abstract

Now that access of large domestic mammals to high-field MRI becomes more common, techniques initially implemented for human patients can be used for the structural and functional study of the brain of these animals. Among them, susceptibility-weighted imaging (SWI) is a recent technique obtained from gradient echo (GE) imaging that allow for an excellent anatomical tissue contrast and a non-invasive assessment of brain iron content. The goal of this study was to design an optimal GE SWI imaging protocol to be used in dogs undergoing an MRI examination of the brain in a 3-Tesla scanner. This imaging protocol was applied to *ex vivo* brains from four dogs. The imaging protocol was validated by visual inspection of the SWI images that provided a high anatomical detail, as demonstrated by their comparison with corresponding microscopic sections. As resolvable brain structures were labeled, this study is the first to provide an anatomic description of SWI images of the canine brain. Once validated in living animals, this GE SWI imaging protocol could be easily included in routine neuroimaging protocols to improve the diagnosis of various intracranial diseases of dogs, or be used in future comparative studies aiming at evaluating brain iron content in animals.

## Introduction

Large animals are increasingly viewed as valuable models for studying human neurological diseases (Youssef et al., 2016; Eaton and Wishart, 2017). Domestic mammals (e.g., dog, cat, sheep, horse) can spontaneously develop brain disorders similar to human ones, and are thus assumed to be more relevant than rodent models with induced diseases (Mcfarlane, 2007; Dickinson et al., 2010; Karageorgos et al., 2011; Chang et al., 2012; Chambers et al., 2015; Schmidt et al., 2015). Another major advantage of large animals is the possibility of non-invasively monitoring the disease with the same MRI scanners as those used in medical facilities, making it easy to translate imaging biomarkers from the animal to the human patient. The recent use of these large models in translational neuroimaging has benefited greatly from the growing access of animals to high-field MR systems and technologies (diffusion- and perfusion- weighted MRI, MR spectroscopy) (Gray-Edwards et al., 2014; Stuckenschneider et al., 2014; Anaya García et al., 2015; Lee et al., 2015; Hespel and Cole, 2018; Johnson et al., 2019; Pieri et al., 2019; Schmidt et al., 2019). Useful in research, these MRI techniques also offer promising ways of improving the diagnosis of various intracranial disorders in veterinary practice (McConnell et al., 2005; Rossmeisl et al., 2007; Carrera et al., 2016; Mizoguchi et al., 2017; Sievert et al., 2017).

Susceptibility-weighted imaging (SWI) is a recent improvement on multiple-echo gradient echo imaging that allows for excellent anatomical tissue contrast and the non-invasive assessment of brain iron content. Anatomical structures that are difficult to see on MRI signal magnitude images can be easily identified on SWI images, owing to the contrast provided by the MRI signal phase images. High-pass filtering of these phase images eliminates unwanted low spatial frequency components. SWI, which uses the filtered phase as a mask, is very sensitive to compounds that distort the local magnetic field. This makes it useful for detecting blood products and calcium, as well as intracerebral iron and therefore microbleeds. It is also well-suited to assessing veins, as deoxyhemoglobin causes both a loss of amplitude and a phase shift (Haacke et al., 1997, 2004; Wu et al., 2012a,b).

In human patients, SWI has mainly been used to detect and monitor (i) microbleeds in traumatic brain injuries (Liu et al., 2012, 2015), (ii) oxygen saturation changes in stroke and intracranial hypertension (Broderick et al., 1993; Haacke et al., 1997, 2010; Plantinga et al., 2014), and (iii) iron accumulation in neurodegenerative diseases such as Parkinson's disease, multiple sclerosis, Huntington's disease, and neuroferritinopathy (Haacke et al., 2004; Martin et al., 2008; Péran et al., 2010; Langkammer et al., 2012, 2016; Liu et al., 2015).

This technique has also been performed in dogs to monitor traumatic brain injury, detect hemorrhage, or identify intracranial venous abnormalities (Noh et al., 2019; Weston et al., 2020; Wolfer et al., 2021). However, no atlas of the canine brain structures revealed by SWI is currently available, even though it is essential for translational research and in veterinary practice.

The present study took place in three stages.

First, given the potential value of SWI in both veterinary practice and translational neuroimaging, and the anatomical contrasts it can provide, we developed an SWI protocol and applied it to four *ex vivo* dog brains. Second, we performed a histological analysis of one of these four brains. Third, we compared the histological sections with the SW images of the same dog brain, with the aim of accurately identifying anatomical components that are usually difficult to view on conventional anatomical MRI (T1/T2/FLAIR).

## Materials and methods

### Animal sampling

The canine brains were collected from four male beagles euthanized under deep anesthesia for teaching purposes (preparation of embalmed cadavers). The experimental procedures related to the preparation of embalmed cadavers were approved by the Animal Ethics Committee of the National Veterinary School of Toulouse (authorization no. 21559-2019071917392588v3). After euthanasia, the head was separated from the body in order to be perfused *via* the common carotid arteries with a rinsing solution (NaCl; flow rate: 15 mL/min, perfusion time: 5 min) and a fixative solution (10% formalin solution, 15 mL/min, perfusion time: 5 min). The heads were then stored in containers filled with 10% formalin solution for 10 months. After this period of fixation, the brain was removed from the skull for *ex vivo* MRI.

### *Ex vivo* susceptibility-weighted imaging

The MRI examination was performed at the Toulouse Institute for Brain Sciences, using a high-field 3.0 T MRI scanner (Intera Achieva; Philips, Best, The Netherlands) with a solenoid coil for signal reception (ø = 8 cm). Twenty-four h before MRI acquisition, the brains were rinsed with water and submerged in a saline solution. Immediately prior to acquisition, they were each placed in an MRI-compatible container (zip-locked hermetic plastic bag) totally filled with saline solution. After being gently agitated in order to manually remove air bubbles, each bag was closed and placed in a foam mold. The space between the bag and the inside of the mold was filled with cotton balls to prevent the bag moving during acquisition (Shatil et al., 2016). SWI acquisition was performed with the following parameters: repetition time = 63 ms, first echo time = 7.5 ms, echospacing = 9.0 ms, flip angle = 23°, field of view (FH x AP x RL) = 49 x 110 x 110 ms, acquisition voxel = 0.35 x 0.35 x 0.35 mm, number of slices = 314, slice orientation = axial strict, NO-SENSE, technique = 3D, number of excitations = 8, total acquisition time = 1 h 32 s.

### Image analysis: Susceptibility weighted imaging

Data pre-processing was performed with Matlab R2019a with the STI Suite Package (Liu et al., 2015). Rigid registration was performed between each of the T2^*^-weighted volumes and the T2^*^-weighted volume at first echo. The transformations were applied to the phase images. The SWI calculation consisted in applying digital high-pass filtering to the phase imaging for each echo time. Low-frequency fluctuations were then eliminated. The resulting phase image was filtered. We then created a phase mask to scale the data over a range of 0–1, in order to highlight tissues with different susceptibilities. The magnitude image of each echo was digitally multiplied by this phase mask three times until significant tissue information was obtained. Minimum intensity projections of the SW images as a function of the number of 3D images for each echo were performed.

### Histological processing

Histological slides were prepared from one of four canine brains previously used for SWI by NeuroScience Associates (Knoxville, TN, USA). The whole brain was embedded and entirely freeze-sectioned into 50-μm slices in the transversal plane. Weil-myelin staining, which highlights white matter, was performed for the whole dog brain, on every 20th section. Details concerning embedding, sectioning, and staining of the canine brain are available elsewhere (Palazzi, 2011).

### Qualitative analysis

A coarser scale reconstruction of the scanned histological data was performed to obtain an interpolated histological volume. Rigid registration of the histological volume onto the SWI volume was performed. Finally, seven cross-sectional SW images were selected to specifically analyze the visual appearance of the telencephalon, diencephalon, midbrain, and rhombencephalon structures.

For all these sections, the location and appearance of the brain structures were compared with those of the histological slides. All the brain structures that could be identified on both the SW images and the histological slides were labeled in accordance with the *Nomina Anatomica Veterinaria* (World Association of Veterinary Anatomists, 2017).

## Results

**Figure 1:**
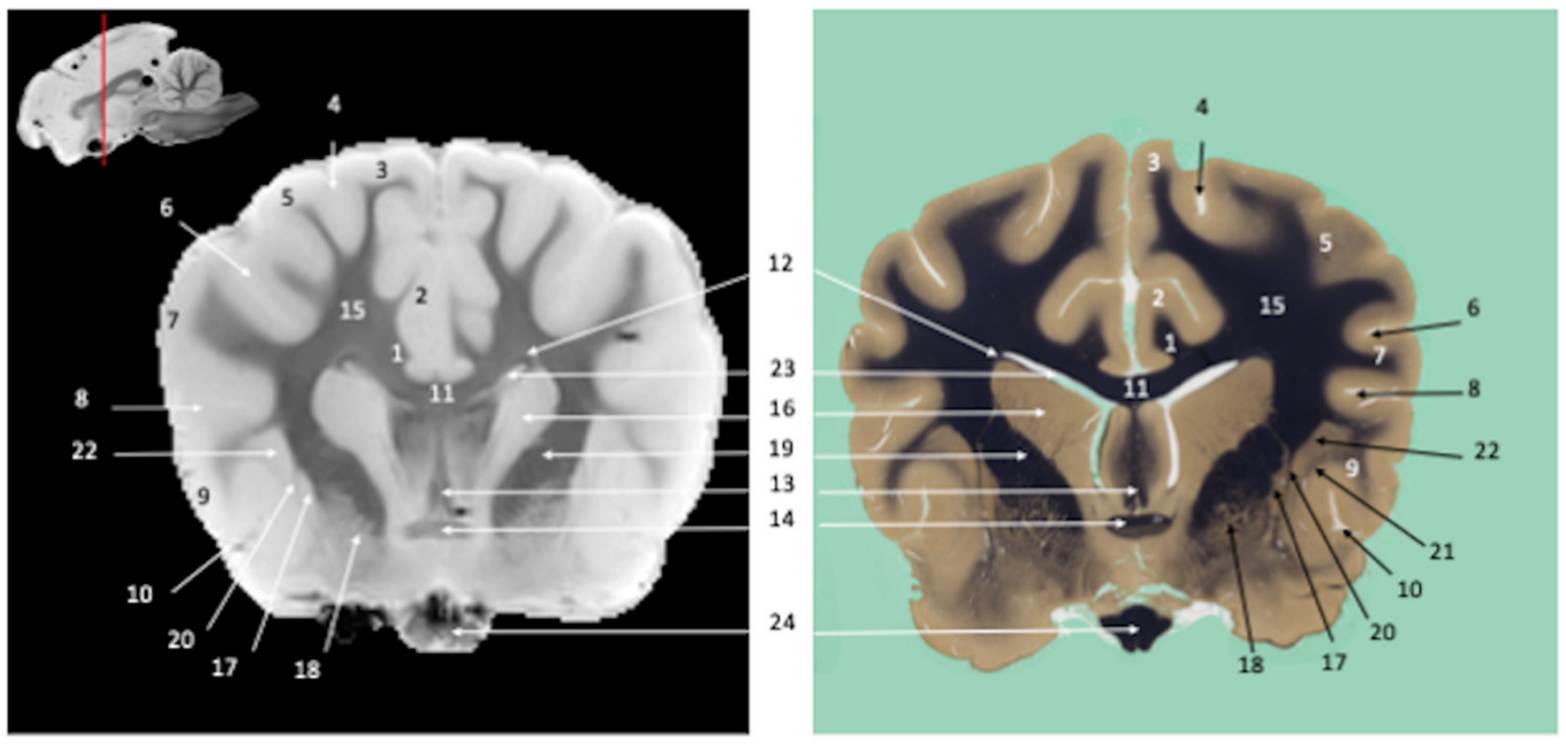
Tranverse section of the telencephalon at the level of the corpus striatum. Susceptibility-weighted images of the canine brain (left) and corresponding histological slide, Weil myelin staining (right). **TELENCEPHALON (1 – 23):** 1, Cingulum; 2, Cingulate gyrus; 3, Marginal gyrus; 4, Marginal sulcus; 5, Middle suprasylvian gyrus; 6, Middle suprasylvian sulcus; 7, Middle ectosylvian gyrus; 8, Middle ectosylvian sulcus; 9, Rostral sylvian gyrus; 10, Rhinal fissure; 11, Corpus callosum; 12, Subcallosal bundle; 13, Fornix; 14, Rostral commissure; 15, Centrum semiovale; 16, Caudate nucleus; 17, Putamen; 18, Globus Pallidus; 19, Internal capsule; 20, External capsule; 21, Extreme capsule; 22, Claustrum; 23, Lateral ventricle. **CRANIAL NERVES**: 24, Optic chiasm.

**Figure 2:**
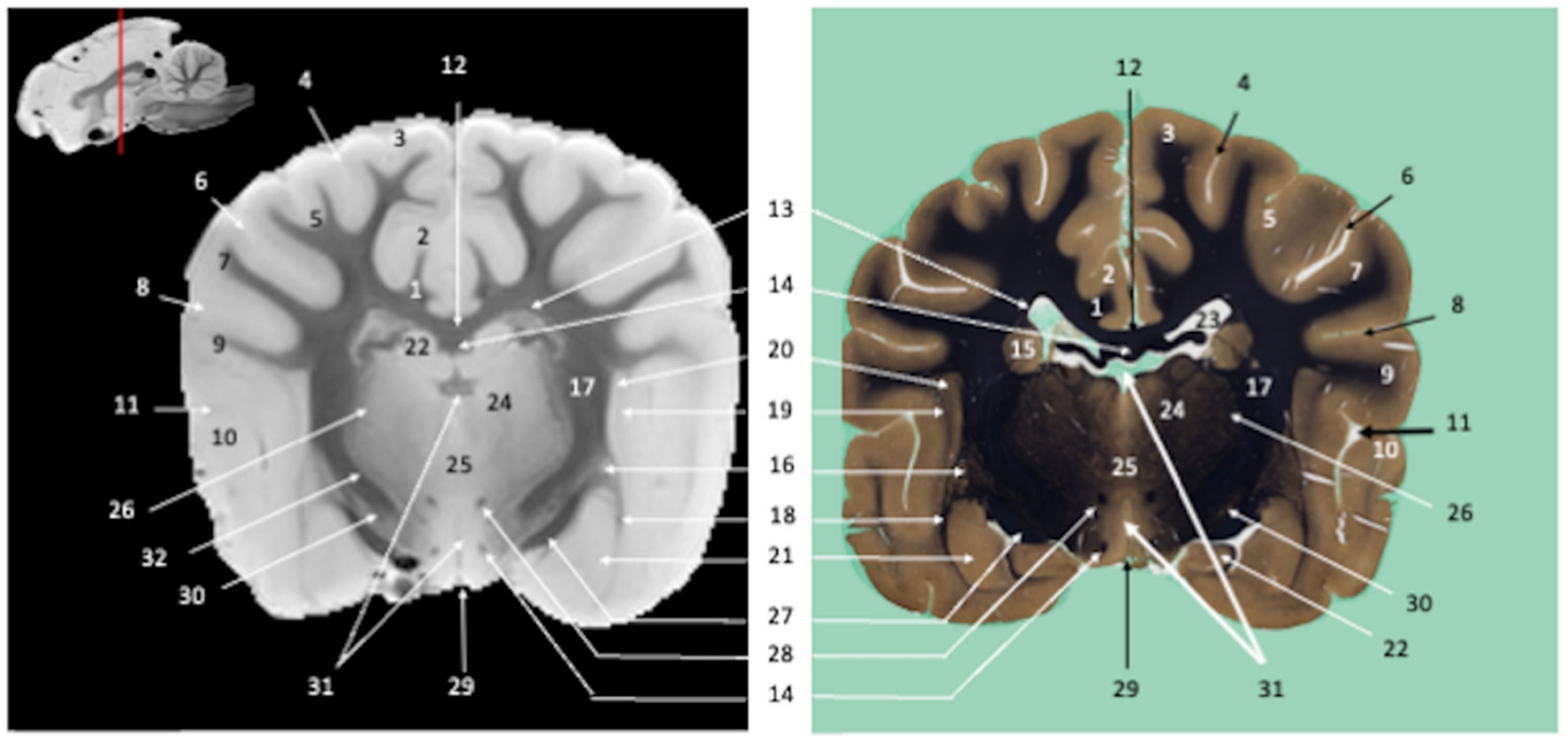
Transverse section of the diencephalon at the level of the infundibulum. Susceptibility-weighted images of the canine brain (left) and corresponding histological slide, Weil myelin staining (right). **TELENCEPHALON (1–23):** 1, Cingulum; 2, Cingulate gyrus; 3, Marginal gyrus; 4, Marginal sulcus; 5, Middle suprasylvian gyrus; 6, Middle suprasylvian sulcus; 7, Middle ectosylvian gyrus; 8, Middle ectosylvian sulcus; 9, Rostral sylvian gyrus; 10, Caudal sylvian gyrus; 11, Pseudosylvian fissure; 12, Corpus callosum; 13, Subcallosal bundle; 14, Fornix; 15, Caudate nucleus; 16, Putamen; 17, Internal capsule; 18, External capsule; 19, Extreme capsule; 20, Claustrum; 21, Amygdaloid body; 22, Hippocampus; 23, Lateral ventricle. **DIENCEPHALON (24–31):** 24, Thalamic nuclei; 25, Interthalamic adhesion; 26, Reticular thalamic nucleus; 27, Optic tract; 28, Mamillothalamic tract; 29, Infundibulum; 30, Endopeduncular; 31, Third ventricle. **MULTIPLE BRAIN REGIONS (32)**: 32, Corticopontine, corticobulbar, and corticospinal projection fibers.

**Figure 3:**
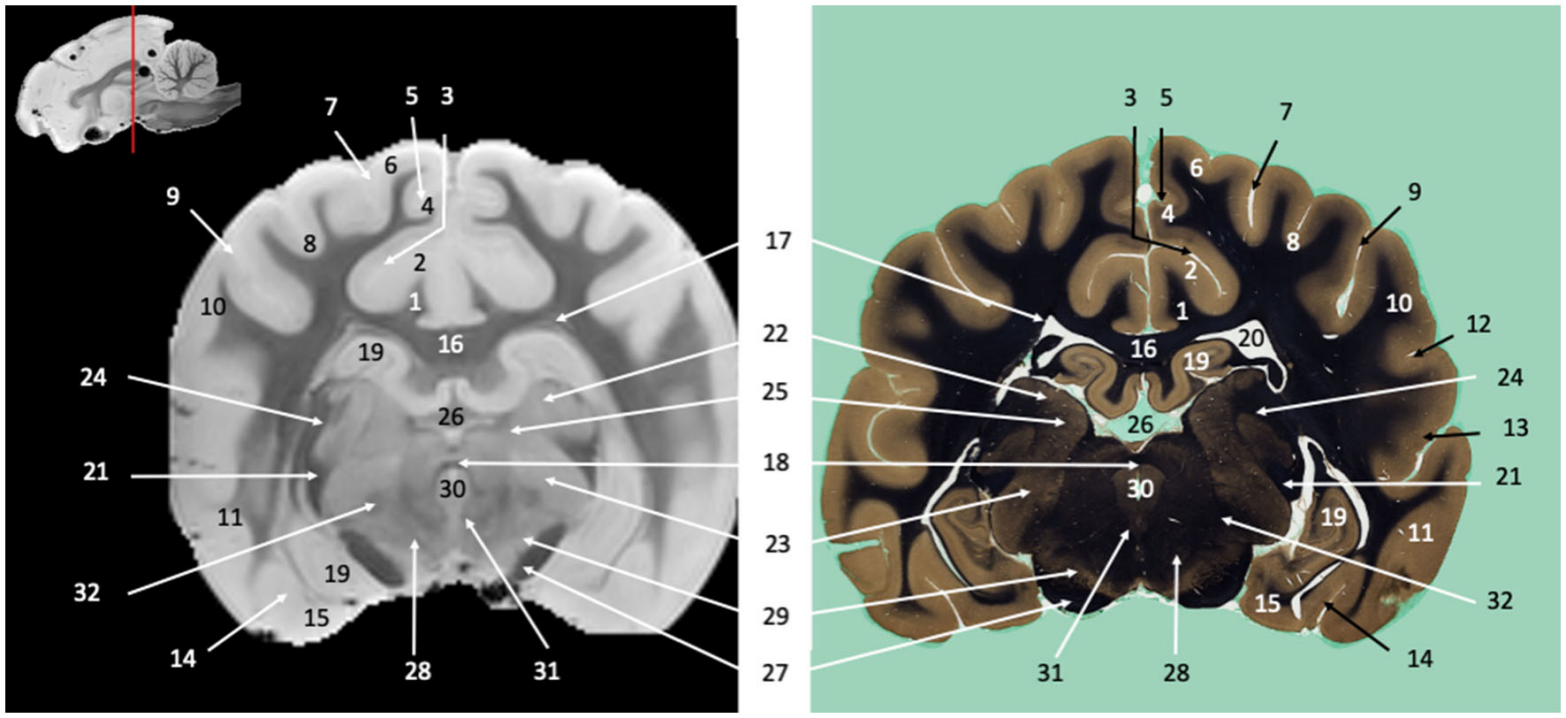
Transverse section of the diencephalon at the level of the geniculate bodies. Susceptibility-weighted images of the canine brain (left) and corresponding histological slide, Weil myelin staining (right). **TELENCEPHALON (1–20):** 1, Cingulum; 2, Cingulate gyrus; 3, Splenial sulcus; 4, Splenial gyrus; 5, Suprasplenial sulcus; 6, Marginal gyrus; 7, Marginal sulcus; 8, Middle suprasylvian gyrus; 9, Middle suprasylvian sulcus; 10, Middle ectosylvian gyrus; 11, Caudal ectosylvian gyrus; 12, Middle ectosylvian sulcus; 13, Caudal ectosylvian sulcus; 14, Rhinal fissure; 15, Piriform cortex; 16, Corpus callosum; 17, Subcallosal bundle; 18, Caudal commissure; 19, Hippocampus; 20, Lateral ventricle. **DIENCEPHALON (21–26):** 21, Optic tract; 22, Lateral geniculate nucleus; 23, Medial geniculate nucleus; 24, Optic radiation; 25, Pulvinar; 26, Third ventricle. **MESENCEPHALON (27–30)**: 27, Crus cerebri; 28, Red nucleus; 29, *Substantia nigra*; 30, Mesencephalic aqueduct. **MULTIPLE BRAIN REGIONS (31–32)**: 31, Medial longitudinal fasciculus; 32, Spinothalamic tract.

**Figure 4:**
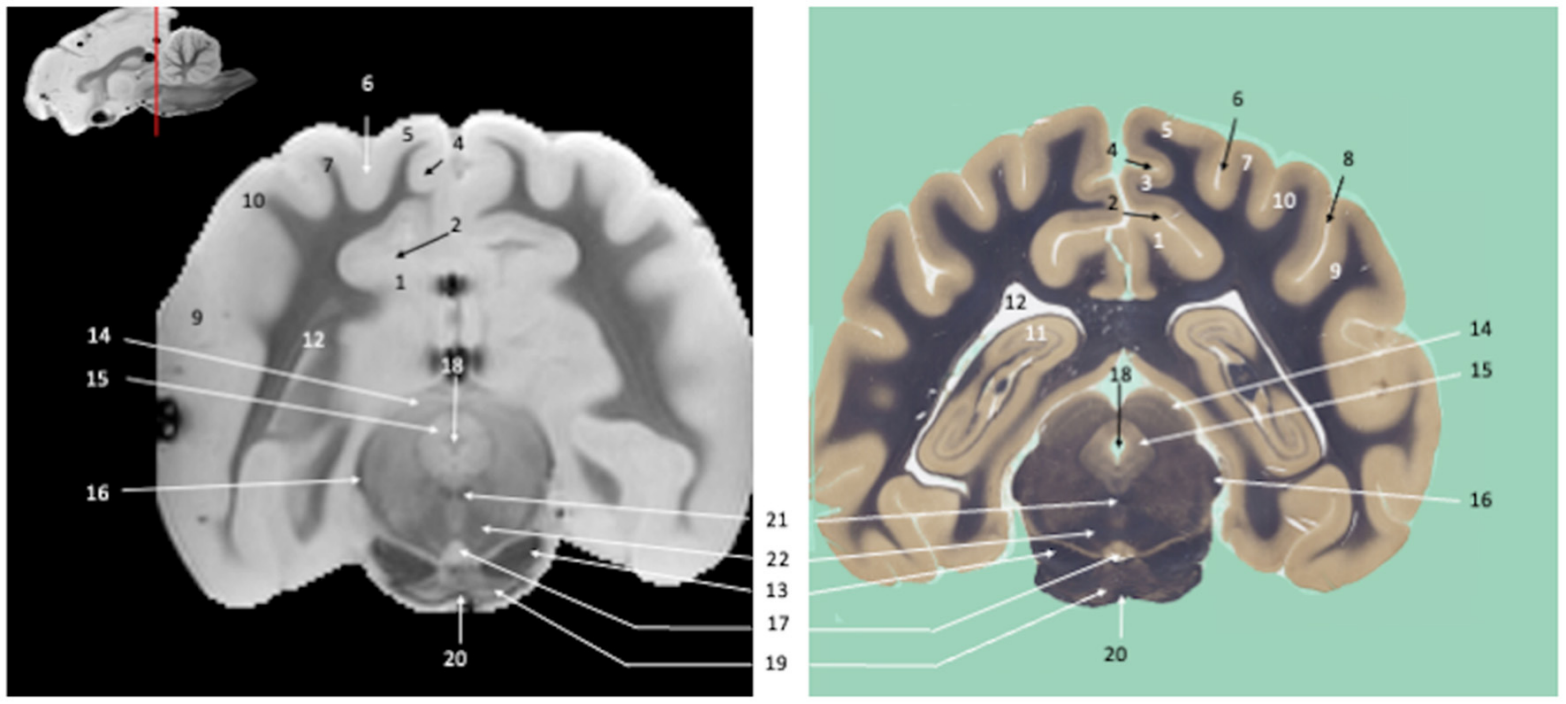
Tranverse section of the mesencephalon. Susceptibility-weighted images of the canine brain (left) and corresponding histological slide, Weil myelin staining (right). **TELENCEPHALON (1–12):** 1, Cingulate gyrus; 2, Splenial sulcus; 3, Splenial gyrus; 4, Suprasplenial sulcus; 5, Marginal gyrus; 6, Marginal sulcus; 7, Ectomarginal gyrus; 8, Middle suprasylvian sulcus; 9, Caudal ectosylvian gyrus; 10, Caudal sylvian gyrus; 11, Hippocampus; 12, Lateral ventricle. **MESENCEPHALON (13–18)**: 13, Crus cerebri; 14, Rostral colliculus; 15, Periaqueductal gray; 16, Brachium of the caudal colliculus; 17, Interpeduncular nucleus; 18, Mesencephalic aqueduct. **RHOMBENCEPHALON (19–20)**: 19, Pontine nuclei; 20, Transverse fibers of pons. **MULTIPLE BRAIN REGIONS (21–22)**: 21, Medial longitudinal fasciculus; 22, cerebellothalamic and cerebellorubral tracts.

**Figure 5:**
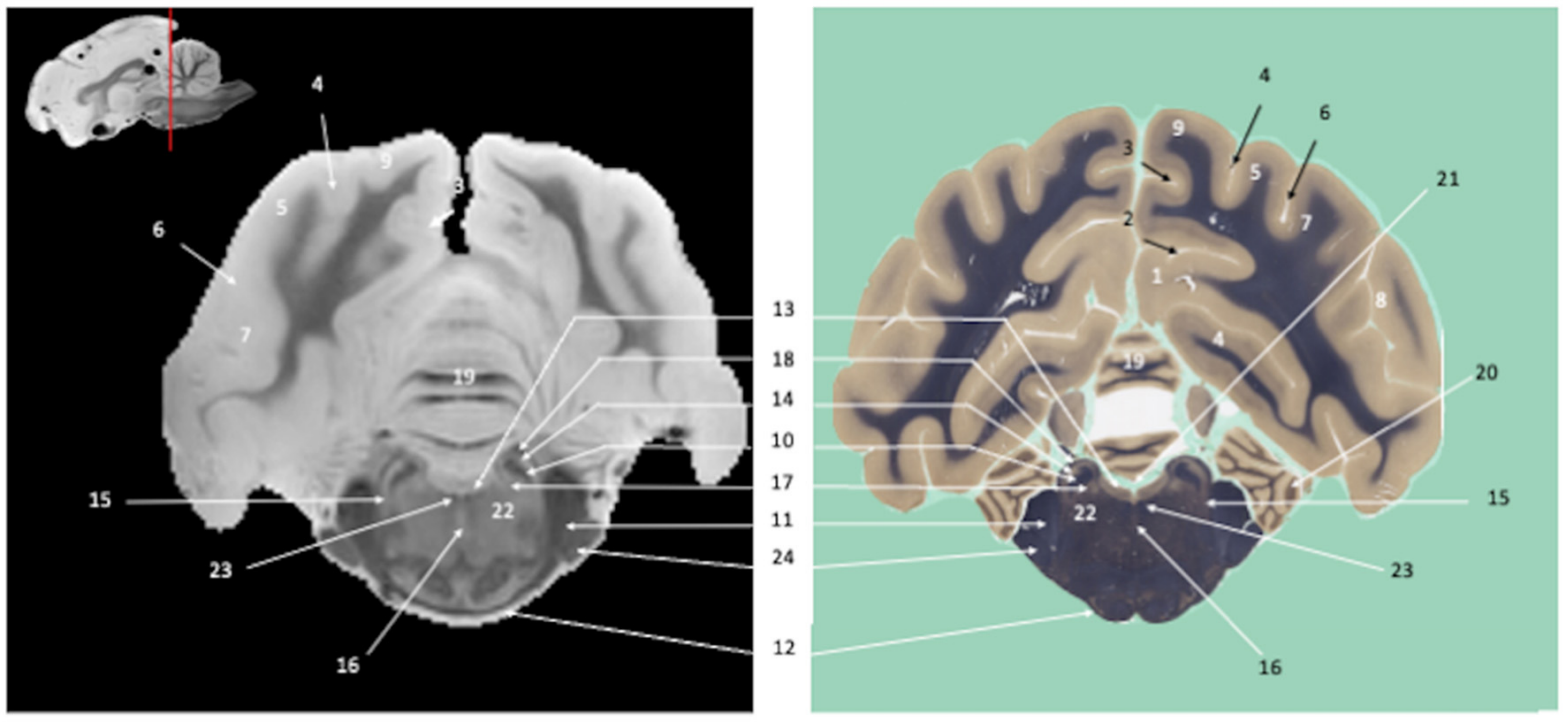
Tranverse section of the metencephalon, at the level of the pons. Susceptibility-weighted images of the canine brain (left) and corresponding histological slide, Weil myelin staining (right). **TELENCEPHALON (1–9):** 1, Cingulate gyrus; 2, Splenial sulcus; 3, Suprasplenial sulcus; 4, Marginal sulcus; 5, Ectomarginal gyrus; 6, Ectomarginal sulcus; 7, Caudal suprasylvian gyrus; 8, Caudal ectosylvian gyrus; 9, Occipital gyrus. **RHOMBENCEPHALON (10–21)**: 10, Rostral cerebellar peduncle; 11, Middle cerebellar peduncle; 12, Transverse fibers of pons; 13, Dorsal tegmental nucleus; 14, Vestibular Nuclei; 15, Pontine nucleus of the trigeminal nerve; 16, Superior central nucleus; 17, *Locus coeruleus*; 18, Ventral spinocerebellar tract; 19, Cerebellar vermis; 20, Paraflocculus; 21, Fourth ventricle. **MULTIPLE BRAIN REGIONS (22–23)**: 22, Reticular formation; 23, Medial longitudinal fasciculus. **CRANIAL NERVES**: 24, Trigeminal nerve.

**Figure 6:**
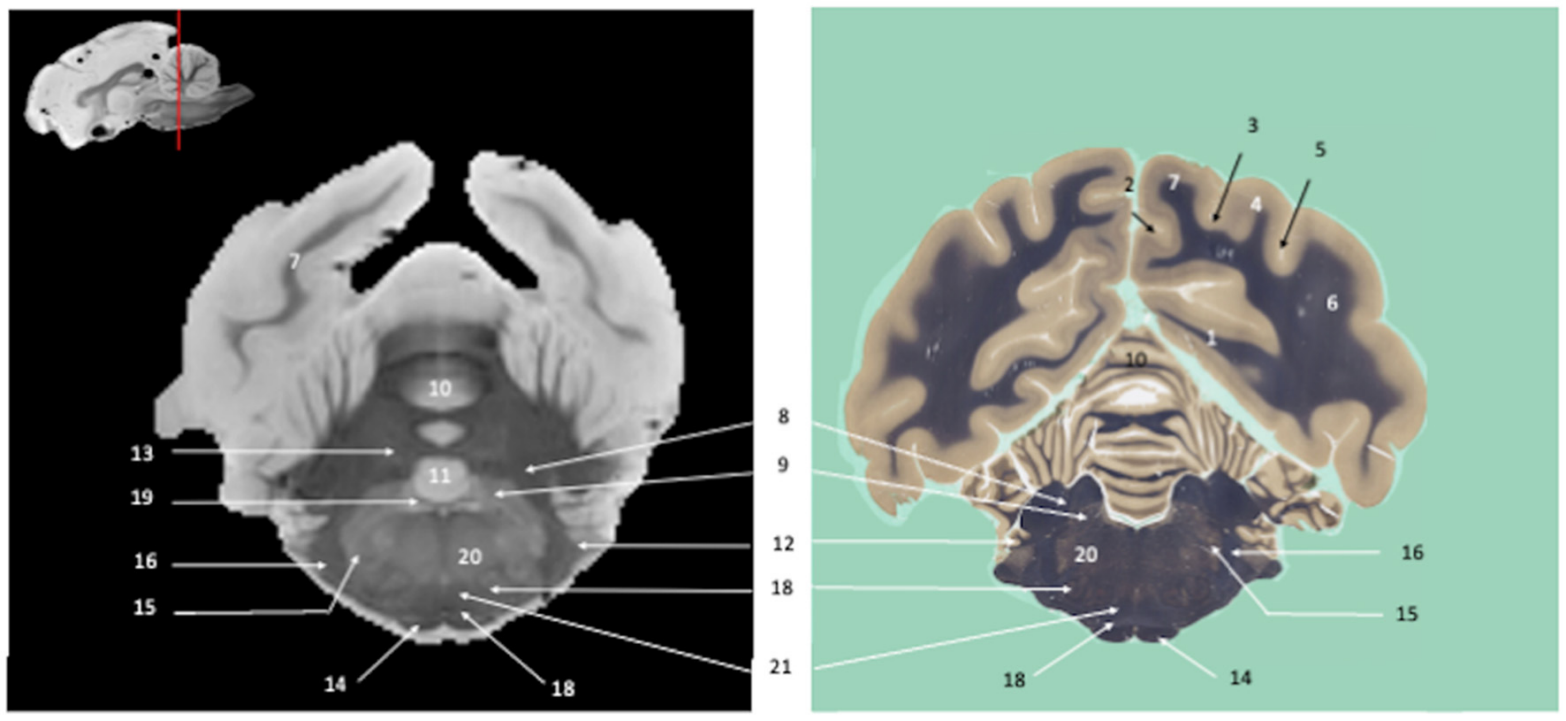
Tranverse section of the myelencephalon and cerebellum, at the level of the genu of the facial nerve. Susceptibility-weighted images of the canine brain (left) and corresponding histological slide, Weil myelin staining (right). **TELENCEPHALON (1–7):** 1, Splenial gyrus; 2, Suprasplenial sulcus; 3, Marginal sulcus; 4, Ectomarginal gyrus; 5, Ectomarginal sulcus; 6, Caudal suprasylvian gyrus; 7, Occipital gyrus. **RHOMBENCEPHALON (8–19)**: 8, Caudal cerebellar peduncle; 9, Vestibular Nuclei; 10, Cerebellar vermis; 11, Lingula; 12, Flocculus; 13, Fastigial cerebellar nucleus; 14, Pyramidal tract; 15, Nucleus of the spinal tract of the trigeminal nerve; 16, Spinal tract of the trigeminal nerve; 17, Superior Olivary nucleus; 18, Trapezoid body; 19, Genu of facial nerve. **MULTIPLE BRAIN REGIONS (20–21)**: 20, Reticular formation; 21, Medial lemniscus.

**Figure 7:**
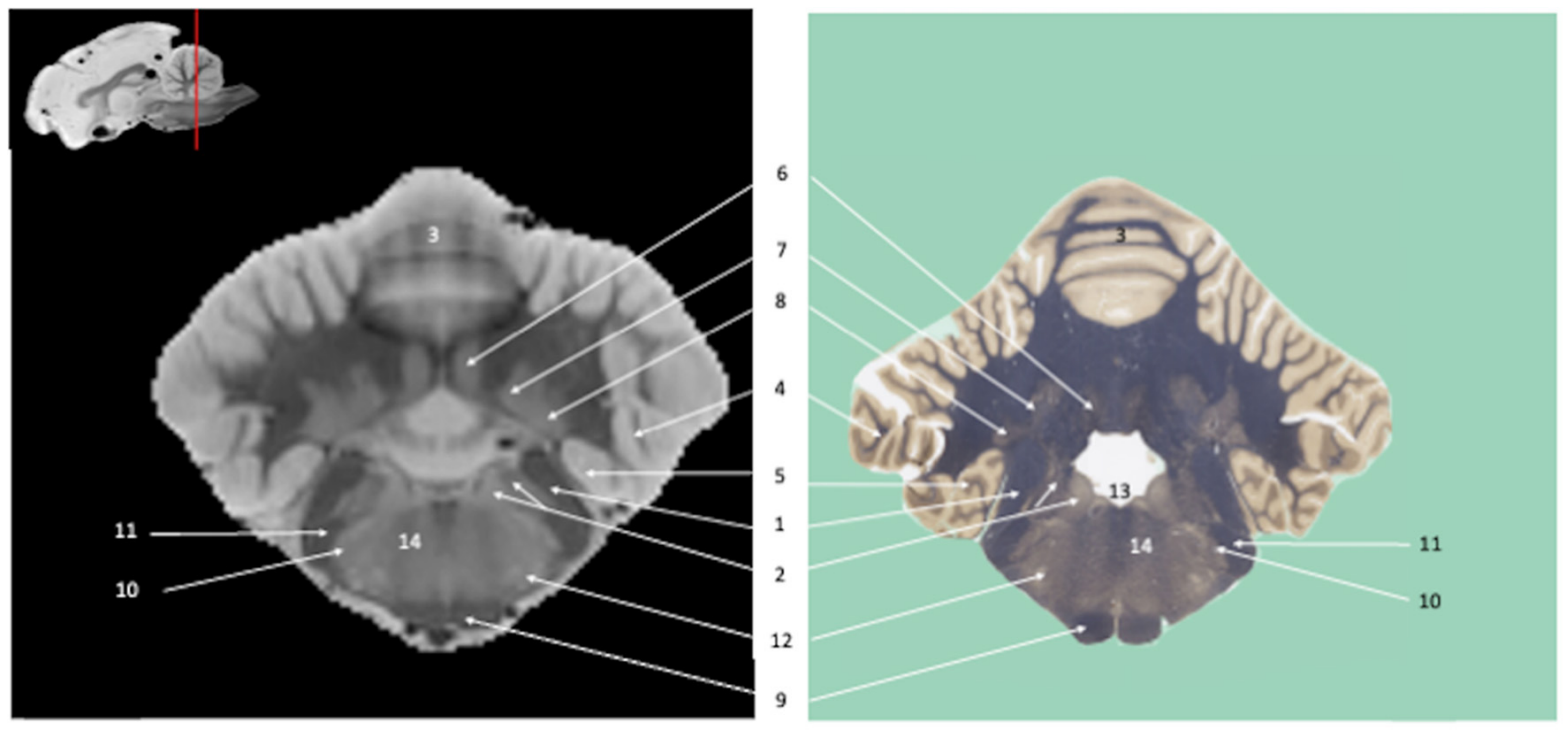
Tranverse section of the myelencephalon and cerebellum at the level of cerebellar nuclei. Susceptibility-weighted images of the canine brain (left) and corresponding histological slide, Weil myelin staining (right). **RHOMBENCEPHALON (1–13)**: 1, Caudal cerebellar peduncle; 2, Vestibular Nuclei; 3, Cerebellar vermis; 4, Ansiform lobule; 5, Paraflocculus; 6, Fastigial cerebellar nucleus; 7, Interpositus cerebellar nucleus; 8, Dentate cerebellar nucleus; 9, Pyramidal tract; 10, Nucleus of the spinal tract of the trigeminal nerve; 11, Spinal tract of the trigeminal nerve; 12, facial motor nucleus; 13, Fourth ventricle. **MULTIPLE BRAIN REGIONS:** 14, Reticular formation.

*Ex vivo* SWI MRI acquisition of the canine brains was successfully performed. The SWI images are displayed in Figures 1–**7**, where their corresponding slicing levels are indicated in the median view of the brain in the upper corner of each image. Qualitative analysis showed that SW images resolved the vast majority of brain structures identifiable on the histological slides, thanks to good resolution and an excellent contrast between white matter (low magnetic susceptibility) and gray matter (high magnetic susceptibility). These structures were labeled, numbered and classified according to the main brain regions to which they belonged.

Visual inspection of the SW images revealed small variations in magnetic susceptibility throughout white matter in canine brains. These images allowed us to identify small white-matter structures, such as the external capsule in Figure 1 (no. 20), the columns of fornix and the mamillothalamic tract in Figure 2 (nos. 14 and 28), the spinothalamic tract in Figure 3 (no. 32), the cerebellothalamic and cerebellorubral tracts in Figure 4 (no. 22), the medial lemniscus and the medial longitudinal fasciculus in Figures 5, 6 (nos. 23 and 21, respectively). Similarly, some gray matter structures of the brainstem, usually hardly delineated on conventional MR images, are more easily identified on SWI images. The examples in our study include nuclei of cranial nerves (e.g., nucleus of the spinal tract of the trigeminal nerve and vestibular nuclei, nos. 15 and 2, respectively in Figures 6, 7) and diverse suprasegmental nervous centers (e.g., interpeduncular nucleus—no. 17 in Figure 4, dorsal tegmental nucleus, superior central nucleus, *locus coerulus*—nos. 13, 16, 17 in Figure 5 and superior olivary nucleus-number 17, in Figure 6).

## Discussion

SWI is a recent MRI technique derived from GE imaging that provides excellent tissue contrast and detects iron in the brain. The images we obtained with our SWI protocol provided high anatomical detail of *ex vivo* canine brains. These sequences could easily be acquired *in vivo*. The signal reserve (high signal-to-noise ratio) and the use of a single coil indicated that it would be possible to use multi-channel coils. The acceleration factor arising from the use of multi-channel coils would allow for a drastic reduction in examination time. This protocol needs to be validated in living animals before it can be used in translational research or veterinary practice.

This is the first study to provide an atlas of the canine brain structures revealed by SWI. The excellent contrast between gray and white matter observed in our SWI images was similar to that described for human brains, with the gray matter exhibiting high magnetic susceptibility, and the white matter low magnetic susceptibility, owing to the diamagnetic nature of the proteins and lipids associated with myelin (Langkammer et al., 2012). As in human brains, variable susceptibility was observed within the white matter, probably owing to differences in myelin content (Evia et al., 2017).

SWI performed using high-pass filtering is very sensitive to the presence of iron, and therefore to microbleeds (Haacke et al., 1997, 2004; Wu et al., 2012a,b). However, phase data, despite their filtering, still possess inherent non-local characteristics associated with environmental magnetic susceptibilities. To circumvent these limitations, quantitative susceptibility mapping (QSM) has recently been proposed as an inverse reconstruction approach, where phase images are mapped onto source images (Deville et al., 1979; de Rochefort et al., 2010; Kressler et al., 2010; Langkammer et al., 2012). It is primarily used to measure iron content (in the form of ferritin, hemosiderin, and deoxyhemoglobin) in brain tissue. Iron quantification provides different and more detailed information than SWI, which simply establishes the presence or absence of iron. Our GE SWI imaging protocol could also provide the necessary methodology to process quantitative susceptibility maps of large animal brains (Supplementary Figure). So far, the evaluation of brain iron metabolism by QSM has never been performed in domestic animals. Yet, such an investigation could prove instructive as brain iron loading is suspected to play a role in the pathological mechanism of Alzheimer's disease (Liu et al., 2018) and domestic mammals, especially the dog, are considered to be potential valuable models of this neurodegenerative disorder (Youssef et al., 2016). Kimotsuki et al. described age-related accumulation of iron in the dog brain highlighted with Perls staining (Kimotsuki et al., 2005). In the present study, a Perls staining was not performed because the dogs were too young to expect accumulation of iron in the brain. Moreover, the long storage (10 months) in formalin can bleach iron from the tissues (Schrag et al., 2010). It will be interesting in a future study to use our SWI protocol to compare QS maps of dogs of different ages and to assess the validity of QSM data by comparing them with Perls stained histological slides.

In addition to providing high anatomical detail, SWI detects hemosiderin, and therefore microbleeds, with high sensitivity. Recent studies carried out in dogs have demonstrated the superiority of SWI over conventional gradient echo sequences in the detection of microbleeds associated with traumatic brain injury (Noh et al., 2019; Weston et al., 2020; Wolfer et al., 2021). SWI could also improve the diagnosis of other intracranial diseases of the dog, such as cerebral amyloid angiopathy (CAA), a disorder characterized by amyloid accumulation in the walls of cerebral blood vessels that can be accompanied by microbleeds. In human patients, SWI has been shown to improve the diagnosis of CAA, compared with T2^*^ GE imaging (Haacke et al., 2007). CAA and parenchymal amyloid deposits are brain lesions of the elderly that are observed in dogs with canine cognitive dysfunction, a syndrome regarded as the canine counterpart of Alzheimer's disease (Ozawa et al., 2016). Combining SWI (to detect possible CAA lesions) with structural MRI and blood biomarkers in cognitively impaired dogs could provide a means of enhancing the diagnosis of canine cognitive dysfunction.

Another advantage of the imaging protocol used in this study is that it can reduce acquisition time (< 45 min). The sequence parameters were optimized for *ex vivo* acquisition: increased number of excitations (*n* = 8), no acceleration factor, and no multi-channel coil. The objective here was to be able to compare MRI acquisition with histochemical imaging. The use of the SWI sequence in a clinical context is entirely feasible, with the same type of signal-to-noise ratio, insofar as it would be performed in an *in vivo* context.

To conclude, this study demonstrates that SWI derived from multiple-echo GE imaging can be successfully applied to the canine brain. Due to the increasing use of the dog in translational neuroscience in recent years, several MRI atlases of the dog have been developed either with the aim of providing stereotaxic reference frame and tissue label for advanced neuroimaging data analysis (Datta et al., 2012; Nitzsche et al., 2019; Johnson et al., 2020; Liu et al., 2020) or to describe relevant canine brain structures as seen on T2-weighted images or turbo spin echo T2-weighted images (Kang et al., 2009; Martín-Vaquero et al., 2011; Jacqmot et al., 2022). The atlas presented in this study is the first to describe brain morphology of the dog as revealed by SWI images with corresponding microscopic sections. The diagnosis of various intracranial diseases of domestic mammals could be enhanced by including SWI in routine neuroimaging protocols. In the context of comparative neuroimaging, this protocol could be used in future large-scale studies assessing brain iron content in animals.

## Data availability statement

The original contributions presented in the study are included in the article/Supplementary material, further inquiries can be directed to the corresponding author.

## Ethics statement

The animal study was reviewed and approved by Animal Ethics Committee of the National Veterinary School of Toulouse.

## Author contributions

GA, SB, PP, GM, and AD designed the study. BC, CM, GM, and AD performed the animal sampling and preparation of brains for *ex vivo* imaging and histological processing. GA and PP designed and optimized the SWI imaging protocol. HG-D and YF acquired the MRI dataset. GA and SB performed the image analyses. AD labeled the SW images and microscopic sections. GA and AD wrote the manuscript. All the authors contributed to manuscript revision and approved the submitted version.

## Conflict of interest

The authors declare that the research was conducted in the absence of any commercial or financial relationships that could be construed as a potential conflict of interest.

## Publisher's note

All claims expressed in this article are solely those of the authors and do not necessarily represent those of their affiliated organizations, or those of the publisher, the editors and the reviewers. Any product that may be evaluated in this article, or claim that may be made by its manufacturer, is not guaranteed or endorsed by the publisher.
