# Supplementary material for: Ex vivo susceptibility-weighted imaging anatomy of canine brain–comparison of imaging and histological sections
Source: Front Neuroanat. 2022 Sep 2;16:948159. doi: 10.3389/fnana.2022.948159 (PMC9481421; doi:10.3389/fnana.2022.948159)
Supplement: Supplementary file 1 [file Data_Sheet_1.docx]

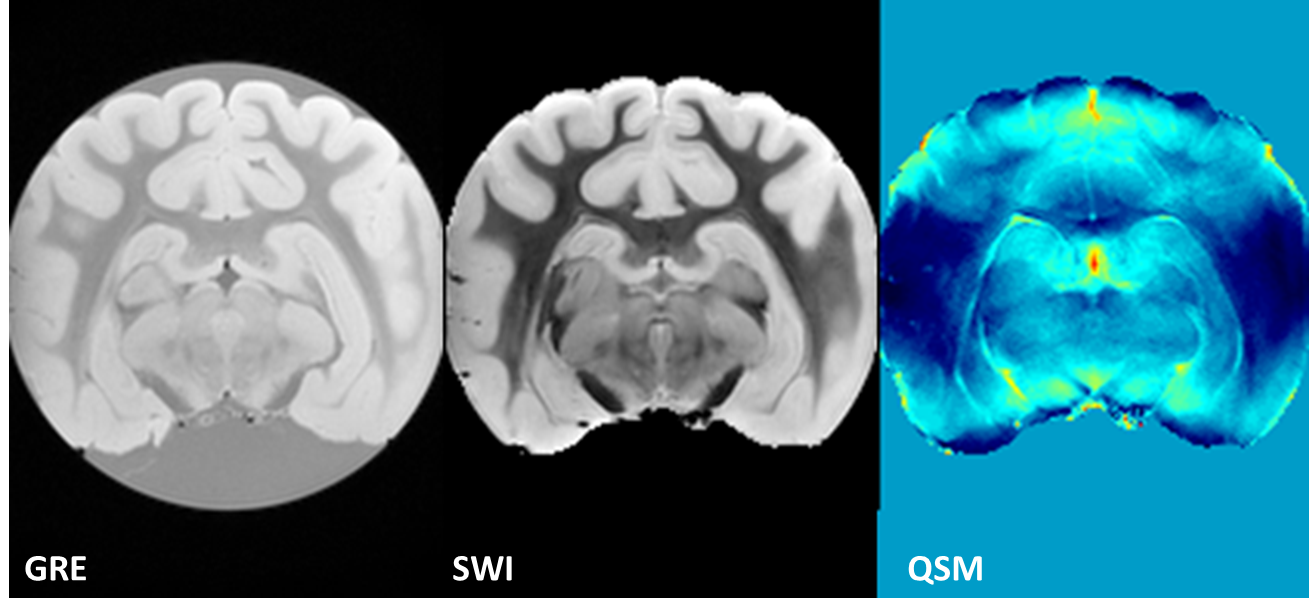


Supplementary Figure: comparison of MRI data. From left to right: gradient echo imaging (GRE), susceptibility weighted imaging (SWI), susceptibility mapping (QSM). Susceptibility imaging is obtained by multiplying magnitude imaging (GRE) and phase imaging (QSM).
